# The economic considerations of patients and caregivers in choice of dialysis modality

**DOI:** 10.1111/hdi.12424

**Published:** 2016-05-15

**Authors:** Rachael C. Walker, Kirsten Howard, Allison Tong, Suetonia C. Palmer, Mark R. Marshall, Rachael L. Morton

**Affiliations:** ^1^ Sydney School of Public HealthUniversity of SydneySydneyNew South Wales2006Australia; ^2^ Hawke's Bay District Health Board, Hawke's BayNew Zealand; ^3^ Centre for Kidney Research, The Children's Hospital at WestmeadWestmeadNew South Wales2145Australia; ^4^ Department of MedicineUniversity of OtagoChristchurch8140New Zealand; ^5^ Baxter Healthcare, (Asia‐ Pacific)ShanghaiChina; ^6^ NHMRC Clinical Trials Centre, University of SydneySydneyNew South Wales2006Australia

**Keywords:** Kidney disease, dialysis, qualitative research, economics, opportunity cost, incentives, reimbursement

## Abstract

**Introduction** Broader adoption of home dialysis could lead to considerable cost savings for health services. Globally, however, uptake remains low. The aim of this study was to describe patient and caregiver perspectives of the economic considerations that influence dialysis modality choice, and elicit policy‐relevant recommendations.

**Methods** Semistructured interviews with predialysis or dialysis patients and their caregivers, at three hospitals in New Zealand. Interview transcripts were analyzed thematically.

**Findings** 43 patients and 9 caregivers (total n = 52) participated. The three themes related to economic considerations were: (i) productivity losses associated with changes in employment; (ii) the need for personal subsidization of home dialysis expenses; and (iii) the role of socio‐economic disadvantage as a barrier to home dialysis. Patients weighed the flexibility of home dialysis which allowed them to remain employed, against time required for training and out‐of‐pocket costs. Patients saw the lack of reimbursement of home dialysis costs as unjust and suggested that reimbursement would incentivize home dialysis uptake. Social disadvantage was a barrier to home dialysis as patients’ housing was often unsuitable; they could not afford the additional treatment costs. Home hemodialysis was considered to have the highest out‐of‐pocket costs and was sometimes avoided for this reason.

**Discussion** Our data suggests that economic considerations underpin the choices patients make about dialysis treatments, however these are rarely reported. To promote home dialysis, strategies to improve employment retention and housing, and to minimize out‐of‐pocket costs, need to be addressed directly by healthcare providers and payers.

## Introduction

As the financial burden of treatment of end‐stage kidney disease (ESKD) on global health systems increases,^1^, ^2^, ^3^ policymakers are challenged to provide widely available, affordable, and quality dialysis. The number of people treated with dialysis in the United States (US) now exceeds 449,000 with the vast majority (91%) being treated with hospital or facility‐based hemodialysis.^4^ One strategy to reduce the cost of dialysis is to increase the proportion of people treated with home‐based peritoneal or hemodialysis^5^, ^6^ given the generally good outcomes and lower cost of these modalities.^7^, ^8^, ^9^

Across the globe, various healthcare reimbursement schemes have been implemented to improve dialysis care and at the same time curtail rising treatment expenditure. There is some limited evidence that macrolevel initiatives can influence dialysis practice patterns.^10^ In contrast, there is less known about microlevel initiatives, and in particular those that deal with patient factors including loss of employment and out‐of‐pocket costs. The extent to which patient contribution influences choice of dialysis modality has not been fully explored.

In New Zealand, home dialysis rates are among the highest in the world with 52% of adults currently receiving home‐based therapy.^4^ Home dialysis is offered to patients with a broad range of socioeconomic backgrounds and is considered a central treatment option for patients who are approaching dialysis. The aim of our study was to describe the perspectives of patients and families on how economic factors such as; costs, benefits, and access to resources, influence decisions about dialysis treatment modality.

## Methods

We followed the Consolidated criteria for reporting qualitative health research (COREQ).^11^

### Context

In New Zealand, patients with ESKD receive all secondary care medical treatment including dialysis and kidney transplantation via a tax‐funded public health system. Patients treated with dialysis at home are not entitled to reimbursement for personal costs (such as electricity, water or transport), although some individual dialysis units provide patients with an annual lump sum payment toward household utility‐related expenses. Additional costs for home dialysis patients, such as transport to clinic appointments, pharmacy costs, and home dialysis set‐up costs are not routinely covered or reimbursed; however, transport costs for hospital dialysis patients are covered (Supporting Information Table S1). New Zealand residents are entitled to a number of government paid benefits, dependent on factors such as existing chronic disease, disability, unemployment, and annual household income. State housing schemes subsidize accommodation costs for lower income households. In New Zealand, as for many countries, people with ESKD have higher rates of social disadvantage than the general population.^12^

### Participant recruitment and selection

As part of a larger study (“The Home First Study”),^13^ we conducted semi‐structured interviews with patients and caregivers to elicit perspectives about home dialysis. We recruited participants from three dialysis units in New Zealand between July 2014 and January 2015. Participants were eligible for the study if they were adults (aged 18 years and over), were “pre‐dialysis” and had received formal predialysis education regarding renal replacement therapy modalities; or had commenced dialysis within the previous 12 months; or were a family member or caregiver. We used a purposive sampling strategy to include a range of demographic and clinical characteristics (i.e., age, ethnicity, dialysis, modality).^14^ Potential participants were identified and recruited by either the nephrologist or nurse specialists in participating units. The study was approved by the ethics committee at each participating hospital.

### Data collection

We conducted semistructured interviews at the participants’ choice of location (i.e., home, a clinic room at the hospital, or via telephone). The interview guide included questions about the economic influences and implications of dialysis treatment choices, that were developed based on a review of relevant literature^15^, ^16^, ^17^ and discussion among the research team (Supporting Information Table S2). Author RCW, a clinician with experience in qualitative interviewing techniques, conducted all interviews. Interpreters were used for three participants for whom English was their second language. Participant recruitment ceased when data saturation was achieved, that is, when no new concepts emerged in subsequent interviews.^14^ We took field notes during interviews, and recorded and transcribed all interviews.

### Data analysis

The transcripts were entered into the software HyperRESEARCH, version 3.7.2 (ResearchWare Inc) for qualitative data management. We used thematic analysis to identify patterns and themes within the interview data and an inductive approach to analyze the data.^18^, ^19^ For the current study, we examined the data for economic concepts such as incentives and reimbursements, as well as respondent considerations about efficiency and equity—for example, the influence of individual patient characteristics such as educational attainment that might help explain differences in attitudes toward home dialysis. RCW coded the transcripts line‐by‐line, identified concepts inductively, and grouped similar concepts specific to patient and caregiver perceptions of economic factors that influenced their choice of dialysis modality. RLM also read the transcripts independently to ensure that the themes reflected the full scope of the data collected (investigator triangulation). This preliminary thematic framework was reviewed by all authors. In subsequent iterations, the coding schema was refined through a series of discussions among the investigator team.

## Results

We interviewed 52 participants (patients [n = 43]; caregivers [n = 9]) who ranged in age between 22 and 79 years, and 25 (48%) were men (Table 1). N (35%) self‐identified as Indigenous Māori and n (27%) as Pacific Island ethnicities. Overall, n (42%) had vocational or university qualifications, while n (28%) had completed primary school (equivalent of elementary school). The median gross household annual income was NZD $31,000–40,000 (USD $19,800‐25,500). N (28%) were in casual, part‐time or full‐time employment.

**Table 1:** Participant characteristics

Characteristics	Patients No. (%)	Caregivers No. (%)
Age (years)		
20–30	3 (7)	0 (0)
31–40	4 (9)	3 (33)
41–50	8 (19)	0 (0)
51–60	10 (23)	1 (11)
61–70	13 (30)	4 (44)
71–80	13 (30)	1 (11)
Ethnicity		
European	10 (23)	4 (44)
Pacific Islander	13 (30)	1 (11)
Maori	15 (35)	3 (33)
Other	5 (12)	0 (0)
Marital status		
Marrried/defacto	25 (58)	6 (66)
Divorced/separated	3 (7)	1 (11)
Single	10 (23)	2 (22)
Widowed	5 (12)	0 (0)
Highest level of education		
Primary school	12 (28)	3 (33)
Secondary school	12 (28)	3 (33)
Certificate or diploma	11 (26)	1 (11)
Degree/higher	8 (19)	2 (22)
Employment status		
Full‐time	9 (21)	0 (0)
Part‐time/casual	3 (7)	2 (22)
Not employed	6 (14)	2 (22)
Beneficiary	18 (42)	3 (33)
Retired	7 (16)	2 (22)
Estimated gross household annual income		
NZ$10–30,000	11 (25)	2 (22)
NZ$31–50,000	12 (28)	3 (33)
NZ$51–70,000	14 (32)	2 (22)
NZ$71–100,000	2 (5)	1 (11)
>NZ$101,000	4 (9)	1 (11)
Number of household occupants		
1–2	23 (53)	4 (44)
3–4	11 (26)	2 (22)
5–6	4 (9)	2 (22)
7–8	2 (5)	1 (11)
9–10	3 (7)	0 (0)
Time to dialysis unit (traveled one way in minutes)		
0–10	10 (23)	2 (22)
11–20	11 (26)	3 (33)
21–40	16 (37)	1 (11)
41–80	1 (2)	0 (0)
>80	5 (12)	3 (33)
Dialysis modality		
Predialysis	18 (42)	2 (22)
Peritoneal dialysis	13 (30)	5 (55)
Home hemodialysis	4 (9)	1 (11)
Facility hemodialysis	8 (19)	1 (11)

NZ = New Zealand; NB = Mean annual income NZ (2014) NZ $93,880.

We identified three themes that described how economic considerations influenced decisions about dialysis treatment: (i) productivity losses associated with changes in employment; (ii) the need for personal subsidization of home dialysis expenses; and (iii) the role of socio‐economic disadvantage as a barrier to home dialysis. Selected illustrative quotations for each theme are presented in Table 2.

**Table 2:** Illustrative quotations for each identified theme

Theme	Quotations
1. Productivity losses related to changes in employment	
Maintaining employment	*“I choose the PD cause then I get back to work fast, the other one they say take months and months to learn, and this one just take one week to learn, too long without work we cannot afford it” (PD5)* *“My daughter and her son don't work, well my daughter is just about to have another baby and my son in law just lost his job so is looking for one now and my wife is on the super, so I'm keeping, trying to keep us afloat here, trying to work and be on dialysis (ICHD7 – changing to HHD))* *“I was thinking about the burden on my husband if I couldn't go back to work” (PD8)* *“I chose the PD cause I think it will be easier to hide from an employer, I can do it at night, and no‐one needs to know, cause really, who wants to employ someone on hemo” (Pre‐d13)* *“Once you get yourself settled on home and back on track you can go and work part time and get an income and that will help” (HHD2)*
Duration of home dialysis training period	*“It was meant to take 5 days but I went through it in 1 day cause I had to go back to work” (PD6)* *“With the other one, you have to move to [city] to learn for 3 months and then I'll lose my job, I don't want to relocate, and that would have a huge impact, for me and the family” (Pre‐d12)* *“If I had to move to do my training for 3 months, that would cost heaps and would be huge impact on every part of our lives” (PD4)*
2. Subsidizing the costs of home dialysis	
Transport costs	*“The problem with me going down to (nearest dialysis unit 2 hours drive) and that and I have to go on my own steam, so I have been paying for that petrol all the time and that's expensive, maybe a hundred dollars every second month, doesn't sound like much but when you're not working at the moment it is” (PD5)* *“They save on the taxi, and they save on the nurse, and they save on your prescription. So we should get our prescription and some taxi to clinic. I think a lot of people worry about it”(HH3)* *“We have to treat everyone fairly, if we go back to the hospital, thousands of dollars for a single person to look after me, at home I look after myself for 75% of the time, and I come to clinic, where is the assistance? it is really unfair”(PD1)*
Set‐up costs for home dialysis	*“I actually need to get a lazy boy or a seat that reclines fully, I asked them about and they said no we don't think that's covered, so that's something I need to source myself, same like trolleys and trays and things for getting myself connected, those things aren't covered, they don't really tell you what the costs of those things” (HH3)* *“I have to do a lot to the house, and probably more cost than normal cause I'm so isolated, and I need try and sort things like a toilet inside and power to the house, I need to get the house better so it's suitable” (Pre‐d8)*
Consumables	*“When I doing my dialysis at night I need the heater on all night then. But there are no costs for hospital dialysis” (ICHD2)* *“The power bill has gone up a lot because I'm home more and feel the cold” (PD5)* *“Cost was one of the things that put me off home haemo too, there are many additional costs for that, water, power, you need a chair, and extra room, more time off work, more everything from what people say. More trips to hospital” (PD8)*
Hidden costs	*“Because no‐one talks to you about the costs of home dialysis I think you just expect that they can't be very much” (ICH5)* *“They don't talk to you about how much either, they didn't talk at all [about cost] and then you wonder why” (Pre‐d17)* *“My mum just said the power might go up and that was about it, no one mentioned it, and then I started stressing about that” (Pre‐d15)*
3. Socio‐economic disadvantage	
Unsuitable home environment	*“It costs for the power and our house now is too cold, and it free to go to hospital and keep warm on the dialysis” (ICHD3)* *“I know this guy, one of the guys that's there, he's in a two bedroom flat, his house is full and it doesn't seem right dialysing in a full house, I think that for a lot of people you know, that might be looking at it like they don't want to go there cause they don't have the room and separate it, so for me that's ok cause I've got a spare room” (HHD2)*
Home ownership	*“This is the first time someone has talked to me about talking to my landlord, tenant people, these are things now I would seriously think about, I didn't know that was an option” (ICHD2)* *“It's just a housing New Zealand house, and it's freezing, and we want to move somewhere bigger and with heating, and so we don't want to ask about a dialysis machine going in the house, cause then they might not move us” (Pre‐d15)*
Inability to afford additional costs	*“My pensions only 13 grand a year, it's not much, I think that more people would do dialysis at home if there was some financial compensation with it” (Pre‐d3)* *“I had to pay for parking and that was $25, and that comes out of a 300 dollar a week benefit” (PD6)* *“I definitely think there should be financial assistance for those on home, definitely, and when you have got more bills, even more so like the water but regardless I think there should be a look into that, and you could see especially for those people who are on benefits and just can't afford anything extra” (HHD1)*
Inability to access financial support	*“We need support about the money stuff, like working out what forms and what your allowed to have, cause they don't tell you, so support with that, (Pre‐d 6)* *“When you don't know the system and there's no one to help, you just don't even know where to start” (Pre‐d13)*

Italicized quotations are from study participants; the codebook containing the themes and sections from each participant coded to the respective themes are available on request.

Pre‐d = Predialysis patient; ICHD = In‐center hemodialysis patient; PD = peritoneal dialysis patient; HHD = home hemodialysis patient.

### Productivity losses related to changes in employment

Patients considered the impact of each treatment on their personal or household productivity when choosing dialysis modality. Many chose home dialysis to maximize flexibility in their dialysis treatment schedule to stay at work or return to the workforce, although the time commitment to train for home dialysis treatments deterred some from choosing home dialysis.

#### Maintaining employment

Some participants felt that their ability to remain at, or return to work was hindered by their dialysis regimen. Some specifically opted for nocturnal home dialysis as the solution to this, as then their daytime hours were not restricted by dialysis. Many were anxious about their employers’ reaction to being on dialysis, and chose a dialysis modality which could remain invisible or undisclosed.

#### Duration of the home dialysis training period

The time required to train for home dialysis and therefore the need for an extended period of time off work was an important consideration in treatment decisions. In particular, some considered whether their employment and resulting income could be maintained during home hemodialysis training, (which can take up to several months), for some, this was a reason they did not choose this treatment. Some participants appreciated their employer's flexibility and support to allow them to commit to home dialysis training. However, those in short‐term or casual employment acknowledged that any sustained time off work could mean a loss of job and household income.

### Subsidizing the costs of home dialysis

Participants established on home dialysis considered the lack of reimbursement for personal treatment‐related costs, such as electricity, water utilities and transport as unfair and inequitable. Participants were aware of the higher cost of facility dialysis to the health system compared to home dialysis, and perceived additional home dialysis‐related expenses as unjust. Many resented the fact that they were personally subsidizing the costs of dialysis to the health system and felt there should be reimbursement schemes or an allowance for home dialysis associated costs. Some participants discussed that having a financial payment for home dialysis might act as an incentive for patients to consider home dialysis.

#### Transport costs

Participants established on home dialysis described the inequity of transport provision compared to those dialyzing in a hospital. They observed facility dialysis patients receiving fully subsidized transport to and from dialysis, and compared this to their own transport costs to training centers, clinic appointments and other check‐ups, for which they did not meet the transport subsidy criteria and, therefore, had to pay themselves. They felt they were being unfairly discriminated against by choosing home dialysis.

#### Set‐up costs for home dialysis

Equipment and home modifications, (for example; tables, hooks, and reclining armchairs) were out‐of‐pocket costs both peritoneal dialysis and home hemodialysis patients were required to pay. Participants considered these costs in their choice of modality, while for others who had not been forewarned, the set‐up costs and additional requirements came as a shock.

#### Consumables

Some home dialysis participants, depending on which renal unit they were from, expressed their concern at having to bear the ongoing costs for dialysis treatment consumables (e.g., fluid, heparin, hand sanitizer). For participants who had previously dialyzed in the hospital they saw no reason why these costs were now their responsibility.

#### Hidden costs

Predialysis patients lacked certainty about the upcoming costs of dialysis. They described a lack of explicit information about additional expenditure and financial support which meant they were not aware of any out‐of‐pocket costs nor how to plan for them. Some home dialysis participants felt that information regarding the additional home electricity costs had purposefully not been shared with them and expressed betrayal by this lack of disclosure, while others were unaware that there would be additional costs.

### Socio‐economic disadvantage

Participants who were socio‐economically disadvantaged described housing constraints as a barrier to home dialysis, either due to the poor or unsuitable housing they lived in or because they did not own their own house. Financial considerations and the inability to afford additional costs of home dialysis were particularly important to those who were socio‐economically disadvantaged. Participants found it difficult to access financial support and navigate social support systems.

#### Unsuitable home environment

For some participants, their housing, often described as damp and cold, was not conducive to home dialysis and the warmth of the facility dialysis units was an inviting break from their own homes. They felt unable to consider home dialysis due to over‐crowding, lack of space for storage of dialysis consumables, lack of a hygienic or private room for dialysis. People who lived in rural compared with metropolitan areas had additional barriers such as an inadequate or unsuitable water supply required for home hemodialysis. For many, relocating to a major city for dialysis was not a feasible option due to a loss of employment, and the potential for additional costs.

#### Home ownership

Participants who were in government or private rental housing felt not owning their own house was a barrier to home‐based dialysis. Some were concerned about approaching their landlord for permission to install a home dialysis system as they feared this may lead to rent increases or eviction. Others had asked and reported that their landlord did not consent to home modifications for dialysis, such as plumbing.

#### Inability to afford additional costs

For socio‐economically disadvantaged participants, financial considerations strongly influenced their decision‐making. Home hemodialysis was described and viewed as more expensive than all other modalities and, therefore, was often the reason it was avoided. Many participants, however, were reluctant to tell health professionals that this was their reason, due to “shame” about their financial position, and instead cited “acceptable” factors such as a phobia for self‐needling as their reason not to dialyse at home.

Facing financial hardship as result of requiring dialysis, the reduced capacity to earn and additional out‐of‐pocket costs was evident for a number of study participants who described their current financial hardship and the constant need for careful budgeting and making sacrifices. Concern about financial hardship resulted in increased stress and pressure on both patients and caregivers. For caregivers, however, the financial implications did not appear to be so critical, as although many still struggled financially, the perceived benefits of home dialysis (i.e. improved patient survival, freedom and flexibility) for their family member, out‐weighed any financial burden.

#### Inability to access financial support

Participants struggled to access financial support both from their dialysis service and government agencies and described difficulty in navigating the social welfare system. Many felt disempowered by the system, and worn down by the need to continually justify their requirement for assistance. For some, the time and expense that was required to gather all the documentation to apply for assistance resulted in them not completing this process and not receiving the assistance to which they were entitled.

## Discussion

Patients considered their potential productivity losses when choosing a dialysis modality, particularly in relation to maintaining or resuming employment to ensure future financial stability. Patients and caregivers believed that it was unfair and inequitable that those on home dialysis personally subsidized the cost of their treatment, while facility dialysis patients did not incur many of these additional out‐of‐pocket costs. Socio‐economic disadvantage was a barrier to home dialysis due to unsuitable housing, the lack of home ownership, and not being able to afford additional out‐of‐pocket costs. Due to feeling ashamed, patients were reluctant to disclose that their personal financial circumstances were driving their decisions.

ESKD more commonly affects those with lower incomes,^20^ and has serious implications for families many of whom are already in socially deprived groups. Our study suggests a lack of transparency regarding the costs of treatment to patients, with some patients being completely unaware of the potential out‐of‐pocket costs for home dialysis at the time of their decision‐making, a factor that may have influenced their choice. In addition, patients felt that they do not receive adequate support to address their financial concerns or issues.

Many participants in our study were aware of the financial savings to the health system of home dialysis and expressed their support for reimbursements to contribute, if not cover their out‐of‐pocket expenses. Based on our findings, we suggest that renal services at a minimum establish and publicise the average out‐of‐pocket costs to patients. If reimbursement for these costs is not available, renal units should develop clear and standardised information to be delivered to all patients at the time of modality decision‐making. Our data suggests that reimbursement of out‐of‐pocket costs directly related to home dialysis might increase the likelihood that some patients and caregivers would consider and be able to afford, home dialysis. A recent international survey indicated significant variation in reimbursement of patient out‐of‐pocket expenses between countries, ranging from full reimbursement of patient out‐of‐pocket expenses in Denmark, as compared to little reimbursement in the United States (US).^21^ Private dialysis providers may contribute to home modification costs associated with home hemodialysis. Although the optimal formula for reimbursement is likely to vary in each setting, it would appear that generally low reimbursement of out‐of‐pocket costs is associated with lower uptake of home dialysis.

The most prominent example of successful incentivization of home dialysis is the US Centers for Medicare & Medicaid Services (CMS) bundled payment system. The initiation of bundling in 2011 has been associated with a 10%–20% increase in home dialysis across various Networks in that country.^4^, ^22^ Another example is Australia, where a government‐funded incentive payment compensates nephrologists for additional work that is required in the planning and management of home dialysis. Initiated in 2005, this scheme is perceived to have had a positive impact on the promotion of home dialysis.^23^ Provider incentives can however have variable and sometimes perverse effects on physician behaviors. It is, therefore, important to ensure that patients still have a choice of treatment, as forcing patients to choose home dialysis would not be successful and may result in increased overall costs and exacerbate treatment inequity.^24^, ^25^, ^26^

Chronic conditions impose significant economic hardship to patients.^27^, ^28^, ^29^ Economic hardship is exacerbated by ineligibility for government support, health service inflexibility, and low health literacy and may lead to patients not being able to effectively choose or engage in self‐management.^30^ This hardship is more pronounced among people from culturally and linguistically diverse or Indigenous backgrounds, and the unemployed. Our results indicate that socio‐economic deprivation is creating a barrier to home dialysis, and potentially only those who can afford home dialysis may benefit from it. This leaves the most vulnerable groups (i.e., low‐income earners, Indigenous populations, and caregivers for family members on dialysis), who may have the greatest gain from home dialysis, without equitable access to it.

A strength of our research is the in‐depth data gained from qualitative interviews that provide detailed insights and understanding of the economic barriers to treatment considered by participants in their decision making. Furthermore, our study included patients who did not have English as their first language, and who are often excluded from similar research. Our study has some potential limitations. Participants were recruited in New Zealand, where dialysis services are all provided through the public health system and, therefore, findings may not be transferable to other countries with private providers, and private‐public funding structures.

Future research should explore interventions that support or incentivize patients and caregivers choice of home dialysis. For example, free financial advice to cancer patients, has been shown to be beneficial in accessing significant additional income for patients and also improving their quality of life and well‐being.^31^ Such studies would be most useful if they were supported by health economic outcome research from a societal perspective, to quantify the effect of interventions on overall resource utilization within any given health system. This perspective is critical to inform health care policy development.

Many countries are looking to decrease the economic burden of treatment for ESKD, and home‐based dialysis may be a potential solution. Our study highlights the economic considerations of home dialysis from the perspective of patients and their caregivers. The implications of these findings need to be understood by healthcare professionals sharing patient and caregiver decision‐making, and by healthcare policymakers and payers.

## Acknowledgments

The authors would like to acknowledge the patients, family members and renal units who participated in this study and all funding received.

## Supporting information

Additional Supporting Information may be found in the online version of this article at the publisher's web‐site:

**Table S1** Comparison of dialysis modality costs in New Zealand.

**Table S2** Exploring patient and carer perceptions and experiences of home dialysis decision making.
