# Supplementary material for: The economic considerations of patients and caregivers in choice of dialysis modality
Source: Hemodial Int. 2016 May 15;20(4):634–42. doi: 10.1111/hdi.12424 (PMC5324572; doi:10.1111/hdi.12424)
Supplement: Supplementary file 1 — Table S1 Comparison of dialysis modality costs in New Zealand. [file HDI-20-634-s001.docx]

|  | Home haemodialysis | Peritoneal Dialysis | Hospital  haemodialysis |
| --- | --- | --- | --- |
| Power to run machine | Yes | Yes | No |
| Water costs | Yes | N/A | No |
| Heating | Yes | Yes | No |
| Transport to clinic appointments | Yes | Yes | Subsidised |
| Set-up costs | Yes | Yes | No |
| Additional pharmacy costs | Yes | Yes | No |
