# Supplementary material for: The economic considerations of patients and caregivers in choice of dialysis modality
Source: Hemodial Int. 2016 May 15;20(4):634–42. doi: 10.1111/hdi.12424 (PMC5324572; doi:10.1111/hdi.12424)
Supplement: Supplementary file 2 — Table S2 Exploring patient and carer perceptions and experiences of home dialysis decision making. [file HDI-20-634-s002.docx]

**Patient interview guide**

**Exploring patient and carer perceptions and experiences of home dialysis decision making.**

1. **Introduction**
   1. Explanation of study, obtain informed consent, demographic info
   2. Can you tell me about what happened when you first found out you had kidney disease?
2. **Information and decisions about dialysis**
   1. When did you first hear about dialysis – what kinds of information or education did you get? *(decision-making process, preferences)*
   2. Who else was involved in your education and decision making – how did they influence your thoughts or decisions about dialysis (their manner, shared decision making?
   3. What sorts of things influenced your beliefs/decisions about the different types of home dialysis (PD, APD, HHD)?
   4. Can you tell me about whether you felt you received enough information to make an informed decision about home haemodialysis or peritoneal dialysis?
   5. If you had to choose again, what sorts of things would help you make decisions about dialysis?
3. **Beliefs about home dialysis**
   1. What were you first thoughts or reactions when you first heard about PD and home HD?
   2. What do you believe are the potential advantages of PD or HHD compared to dialysis in hospital - why?
   3. What do you believe are the potential risks of PD or HHD compared to dialysis in hospital - why?
   4. If someone asked you what dialysis they should choose, what would you tell them?
4. **Living with home dialysis**
   1. Have you considered changing your place or type of dialysis– why?
   2. For you, what do you think are potential reasons that would stop you having home dialysis? For others?
   3. In NZ the number of people on home dialysis has reduced over the last 15 years, why do you think that might be?
5. **Socioeconomic and cultural issues impacting on home dialysis**
   1. Will home dialysis have a financial impact on you or other members of your family - how? (paid work, unpaid work, benefits, expenses e.g. travel, costs, electricity, water)
   2. Do you think there are social or cultural issues that might influence decisions about home dialysis for you or your family?
   3. What are your beliefs/thoughts about financial assistance (i.e. whether adequate and/or accessible); and if it influences their thinking towards home dialysis?
   4. Are there cultural or spiritual factors which influence the place of dialysis for you? (non-medicalised environment, days of rest, whanaunatanga, wairua)
   5. How could the education and support you received about dialysis options better address your cultural or spiritual needs?
6. **Conclusion**
   1. What kinds of support or services would you need if you did dialysis at home – why?
   2. Is there anything else that you think is important to add?
